# Deep Indentation Tests of Soft Materials Using Mobile and Stationary Devices

**DOI:** 10.3390/ma17174233

**Published:** 2024-08-27

**Authors:** Joanna Nowak, Mariusz K. Kaczmarek

**Affiliations:** Faculty of Mechatronics, Kazimierz Wielki University, 85-074 Bydgoszcz, Poland; joanna_n@ukw.edu.pl

**Keywords:** indentation test, mechanical parameters, soft materials, phantoms, objectivity of measurements, mobile and stationary apparatus

## Abstract

Measurements of the properties of soft materials are important from the point of view of medical diagnostics of soft tissues as well as testing the quality of food products and many technical materials. One of the frequently used techniques for testing such materials, attractive due to its non-invasive nature, is the indentation technique, which does not puncture the material. The difficulty of testing soft materials, which affects the objectivity of the results, is related to the problems of stable positioning of the studied material in relation to the indentation apparatus, especially with a device held by the operator. This work concerns the comparison of test results using an indentation apparatus mounted on mobile and stationary handles. The tested materials are cylindrical samples of polyurethane foams with three different stiffnesses and the same samples with a 0.5 or 1 mm thick silicone layer. The study presented uses an apparatus with a flat cylindrical indenter, with a surface area of 1 cm^2^, pressed to a depth of 10 mm (so-called deep tests). Based on the recorded force changes over time, five descriptors of the indentation test were determined and compared for both types of handles. The tests performed showed that the elastic properties of foam materials alone and with a silicone layer can be effectively characterized by the maximum forces during recessing and retraction and the slopes of the recessing and retraction curves. In the case of two-layer materials, these descriptors reflect both the characteristics of the foams and the silicone layer. The results show that the above property of the deep indentation method distinguishes it from the shallow indentation method. The repeatability of the tests performed in the mobile and stationary holders were determined to be comparable.

## 1. Introduction

One of the directions in soft tissue modeling, apart from intensively developed numerical modeling, is the search for synthetic materials called physical models or phantoms, which reflect the properties of soft tissues or organs. Such phantoms are then used in research to better understand the phenomena occurring in tissues or implants, as well as in the education of doctors and physiologists. Indentation tests are among the most frequently used research methods used to determine the mechanical properties of soft materials: gels [1,2], tissues [3,4,5,6,7,8], food [9] and technical materials [10,11,12,13,14,15,16,17,18]. The method of conducting indentation tests may vary due to the following: (i) the size and shape of the indenter used (the diameter of the indenter is usually from a fraction to approximately 10 mm; it has a flat, circular or conical end), (ii) the depth of its immersion in the material (from a fraction to 10 mm), (iii) the type of data being controlled and recorded and (iv) the mobile or stationary mode of operation of the measuring device. In short-term tests performed to assess elastic properties, the maximum force in response to a given depression or a depression at a given force is recorded (in medicine, so-called tonometry). Tests carried out over a longer period are performed for materials with significant internal viscosity. They are carried out by forcing a specific depth and recording the time-varying force of the material’s response to the indenter (relaxation) or by setting a constant force and observing the change in the depth in time (material creep). In addition to the above-mentioned elastic and viscous properties, indentation tests can also test a certain type of material strength by leading to its puncture; see, e.g., [14].

Natural objects or material samples used in indentation testing may vary significantly in thickness and degree of homogeneity. From the point of view of indentation tests, samples with large thicknesses are treated as semi-infinite, e.g., [1]. Thin samples are usually treated as two-dimensional membranes and tested suspended after being mounted on the outer edge, e.g., [12,13]. In other cases, sample thicknesses play a key role in the interpretation of results and tests are most often carried out on rigid substrates, e.g., [6,16]. Most of the results of indentation tests concern materials that are assumed to be homogeneous. Examples of heterogeneous materials tested using the indentation method are layered materials, in particular two-layer materials with one layer much thinner than the other. This mainly applies to biological materials or their phantoms [4,6,7,8].

The mathematical description of indentation tests may be empirical relationships, but more often, simple relationships derived from continuum models of materials or full analytical or numerical solutions of appropriate initial boundary problems [19,20,21,22,23,24,25,26,27,28,29] are used for this purpose. The complexity of modeling based on the concept of continuum media increases when it concerns large deformations, multiphase materials (e.g., gels), inhomogeneous or anisotropic materials, as well as irreversible phenomena such as plastic deformation or cracking. The first approximation in continuum modeling is the linear-elastic material model, while more advanced models for large deformations are the hyperelastic model [6] and the hyper-viscoelastic model, e.g., [17].

In the biomechanical and medical literature, indentation tests are being developed to improve tissue diagnostics, e.g., to identify edematous tissue in the limbs [3,30,31,32,33,34,35,36,37] or swelling of the posterior axillary fold in women after mastectomy [38,39,40], as well as to assess the mechanical parameters of the skin [41]. Mobile devices are particularly convenient for medical applications. In in vivo measurements, they involve an indentation device held by the operator. Taking into account the irregular shape of the body and the possibility of moving the test object, each such measurement is a challenge and requires considerable experience in positioning the indenter in contact with the patient’s body. The results of such studies are characterized by limited repeatability and poor precision and are subject to a subjective factor.

Indentation tests carried out for food materials, polymer foams [9,14,15,16,17,18] or thin flexible membranes [10,11,12,13] are undertaken in order to estimate the mechanical properties of these materials. Then, the results of these tests can be used in the quality control process, selection of the optimal material in production (e.g., mattresses or seat fillings) or selection of protective material during the transport of delicate goods [16].

From the review of the publications presented above, it can be concluded that many works have been written on indentation methods applied to soft materials. Measuring devices and methods of interpreting test results are constantly being developed. Attempts are also made to apply the method to increasingly complex materials or non-elastic ranges of deformations. Nevertheless, the fact remains that an issue that has not been thoroughly researched is the optimal selection of indenter test parameters (type and size of the indenter, indentation depth, registration time, etc.) for testing specific soft materials. In particular, this applies to the parameters of testing heterogeneous materials in the form of a layered system, as soft tissues can be approximated. Another insufficiently understood problem is the assessment of the level of objectivity of the results when using a mobile device or a solution that increases this objectivity.

This work focuses on deep indentation tests in the elastic range (below the puncture threshold). The tested materials are polyurethane foams with three different stiffnesses and the same foams with a silicone layer with a thickness of 0.5 or 1 mm. The geometry and stiffness range of the foams and silicone were selected so that the prepared samples could be treated as phantoms of soft tissues of the limbs (the foam imitates subcutaneous tissue and muscles, while the silicone layer acts as skin). Examples of similar phantoms can be found in the literature for skin layers [42] and lungs [43]. The research procedure proposed in this work uses an indentation apparatus mounted, similarly to the tested object, onto specially designed mobile or stationary holders. The main aim of the work is to compare the results of deep indentation testing for nine (homogeneous and heterogeneous) soft materials and to assess the impact of the method of mounting the device on the values of the proposed set of five descriptors obtained from the result recorded in the indentation test. The discussion addresses the problem of the objectivity of the indentation test results and the impact of the test parameters on the characteristics of soft materials, including those with non-homogeneous layers.

## 2. Materials and Methods

Nine different cylindrical samples made of soft materials were used as the research material. The shape, dimensions and stiffness of the samples were selected in such a way that they approximately correspond to human lower limbs. Three samples were cylinders cut from polyurethane foams with significantly different stiffnesses used in the furniture industry. The names adopted for these samples were S (soft foam), M (semi-rigid foam) and H (rigid foam). The cylindrical samples had the following dimensions, where d—diameter, L—length of the cylinder: S (d = 0.156 m, L = 0.593 m), M (d = 0.155 m, L = 0.6 m), H (d = 0.152 m, L = 0.6 m). The apparent densities of the tested foams were determined using the gravimetric method and were 65.03, 37.74 and 42.07 kg/m^3^, respectively. Assuming that the density of polyurethane was approximately 1200 kg/m^3^, the porosities of the S, M and H foams were 0.946, 0.968 and 0.965, respectively.

The uniaxial stiffness of the tested foams was measured according to the International Organization for Standardization, 2021 [44], in a Zwick Roell Z005 universal testing machine with the TestExpert III data analysis software. For each material, 5 samples with dimensions of 10 cm × 10 cm × 5 cm were prepared and tested. The measurement results are included in Table 1. The obtained Young’s modulus values indicate high heterogeneity in the samples even though they were obtained from the same block. It is also worth adding that the softest foam (S) was characterized by high internal viscosity (sometimes also called the shape memory effect).

The remaining six phantoms were made as cylinders of the same S, M and H foams, adding a layer of 0.5 or 1 mm thick silicone to their side surfaces. To prevent the silicone layer from moving along the foam surface or wrinkling during the indentation test, the silicone was tensioned and glued to the foam along the two abutting edges using a flexible adhesive.

**Figure 1:**
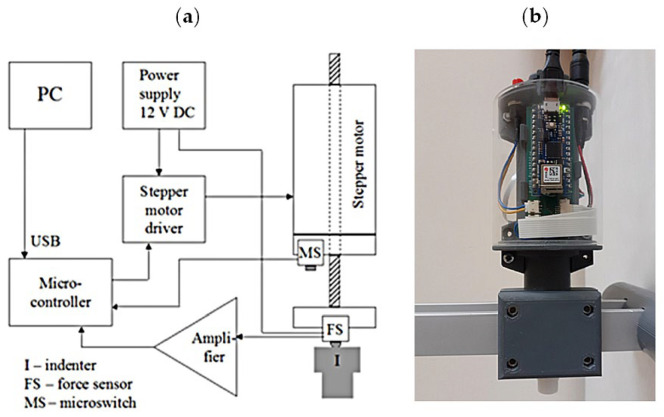
Block diagram of the measurement system (**a**) and a photo of the complete indentation apparatus (**b**).

**Figure 2:**
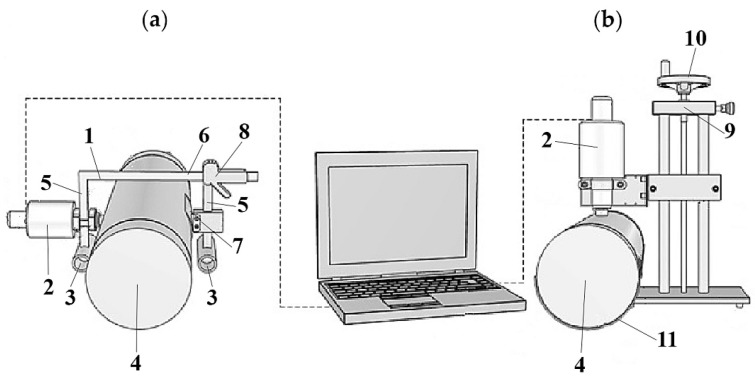
Measurement systems with a mobile (**a**) and stationary (**b**) holder, along with a phantom (4) and the indentation apparatus (2) (a detailed description is given in the text).

Indentation tests of the above-described materials were carried out using an indentation apparatus, the block diagram and view of which is shown in Figure 1, and mounted on movable or stationary holders, as shown in Figure 2. The apparatus and holders were designed and manufactured at the Faculty of Mechatronics (Kazimierz Wielki University in Bydgoszcz, Poland). Additionally, the same materials were examined with a commercial SkinFibroMeter (Delfin Technologies, Kuopio, Finland), held in the operator’s hand.

The indentation apparatus consists of two blocks: the indenter’s drive system and the pressure-force measurement system on the indenter, connected to a microcontroller (Arduino Nano 33 IoT), as shown in Figure 1a. The drive system includes a stepper motor (L2018S0604, 5x1, Nanotec, MotionUSA, Dublin, Ireland) and a motor controller (Pololu 2968, Pololu Corporation, Las Vegas, NV, USA) connected to the digital pins of the microcontroller. The force measurement block consists of a force sensor (FSS020WNGR, Honeywell, Charlotte, NC, USA) connected to an amplifier from which the signal is sent to the analog input of the microcontroller. The force sensor and stepper motor driver share a common 12 V power source. The device communicates with a PC via a USB interface and specialized software created in the LabView environment. Calibration of the force measurement block was performed using a dynamometer (Sauter FL50, Kern & Sohn Gmbh, Ziegelei 1, Balingen, Germany). The flat-ended cylindrical indenter (I) is made of hard plastic and the contact area with the tested material is 1 cm^2^.

Sketches of the complete measurement system with a mobile and stationary holder are shown in Figure 2a,b, respectively. The mobile holder (1) of the indentation apparatus (2) is used for measurements in a horizontal position and its support points (3) lie on the same surface as the tested sample (4). The handle is composed of two vertical guides (5) connected by a horizontal beam (6). An indentation apparatus (2) is mounted on one of the guides, permanently connected to the horizontal beam, which can be moved along the guide, adjusting its position to the height of the measured object. A movable clamp (7) with a profiled clamp is mounted on the second guide to stabilize the position of the tested object. This guide is connected to the horizontal beam via a clutch with a handle and a release button (8), which makes it possible to quickly adjust the guide spacing to the size of the tested object. In the stationary version of the device (Figure 2b), the measurement is carried out in the vertical direction. The indentation apparatus (2) is mounted on a tripod (9) with a manual screw mechanism (10) enabling the apparatus to be moved vertically to bring it closer to the tested object (4). A profiled support (11) is attached to the base of the tripod, ensuring proper positioning of the tested object in relation to the indentation apparatus. In both cases, the supports had a curvature adapted to the radius of the tested phantoms.

In all the tests described in the work, the indenter was placed on the opposite side to the support on which was rested cylindrical phantom, then the sample was penetrated to a depth of approximately 10 mm (deep penetration tests) at a constant speed of 1 mm/s. Then, the position of the indenter did not change for 60 s, and then the indenter was withdrawn at the same speed.

**Figure 3:**
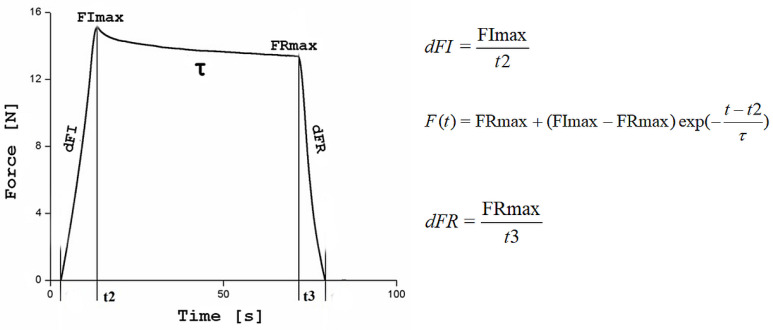
Example curve recorded in the indentation test for H foam with a 1 mm thick silicone layer, with characteristic parameters and formulas necessary to calculate them. dFI and dFR are the slope of the curve during indentation and retraction, FImax and FRmax are the maximum forces at the end of indentation and at the beginning of indenter retraction and τ is the relaxation time.

For each phantom, the tests were repeated 6 times. The reaction force of the material to the indenter was recorded throughout the entire test period. An example of the force–time dependence for one of the tested phantoms is shown in Figure 3.

To compare the test results, a set of five descriptors marked in Figure 3 was selected: linear approximations of the slope of the force during indentation dFI and retraction dFR, maximum force in the loading FImax and retraction FRmax phases and the relaxation time τ, determined from the exponential approximation of the curve recorded for a stationary indenter (relaxation curve). The descriptors were determined using software written in the Matlab environment. The mean values and standard deviations of the descriptors from the 6 tests at different places on the phantom surface were determined for each phantom. The measurement points were arranged in a straight line and the distance between them was 2 cm.

Apart from the tests to a depth of 10 mm with a device mounted on mobile and stationary holders, shallow indentation tests were carried out, to a depth of approx. 1 mm, using the SkinFibroMeter device: https://delfintech.com/products/skinfibrometer/ (accessed: 21 August 2024). The SkinFibroMeter has a stationary, cylindrical, flat-ended indenter with a length and a radius of 1.25 mm, attached to a circular base with a radius of 11.5 mm. During the test, the force acting on the indenter and the base of the apparatus head is measured. Tests using the SkinFibroMeter are carried out using the quick-contact method, which involves determining the average pressure force on the indenter by applying the device head to the tested material five times for approximately 0.5 s.

## 3. Results

**Figure 4:**
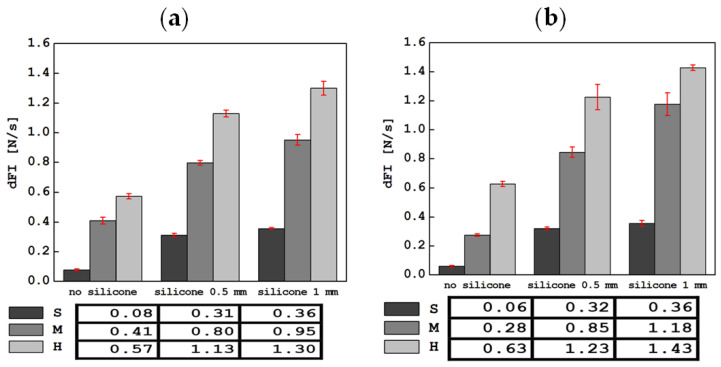
Comparison of average dFI values (slope of the curve in the indentation phase) along with standard deviations for all tested materials in tests with the device in a mobile (**a**) and stationary (**b**) holder.

**Figure 5:**
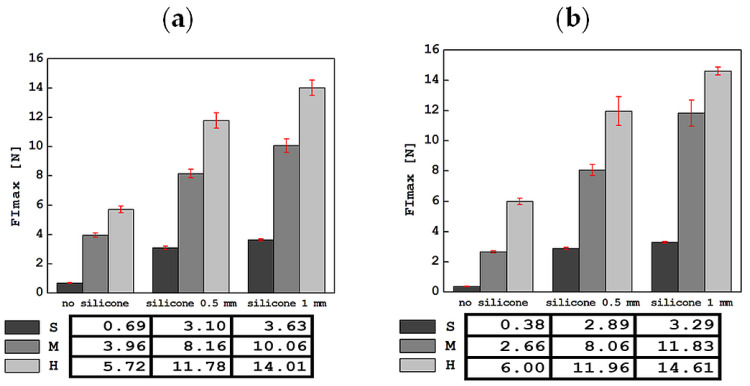
Comparison of the average values of the maximum force in the indentation phase, FImax, along with standard deviations, for all tested materials in tests with the apparatus in a mobile (**a**) and stationary (**b**) holder.

**Figure 6:**
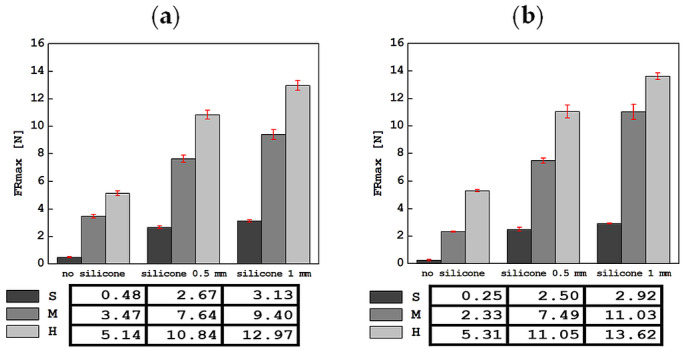
Comparison of average values of the maximum force in the retraction phase, FRmax, along with standard deviations for all tested materials in tests with the apparatus in a mobile (**a**) and stationary (**b**) holder.

**Figure 7:**
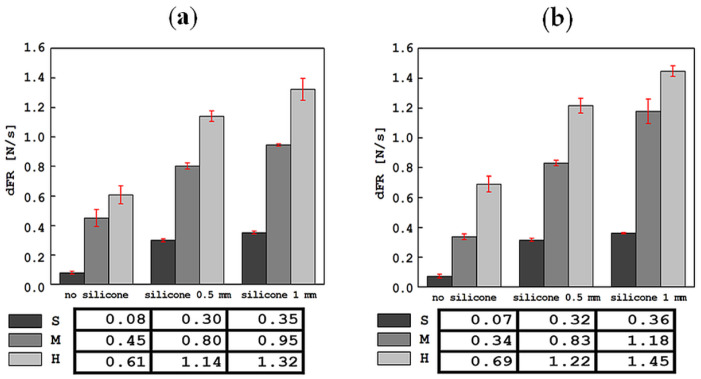
Comparison of average values of the slope of the curve in the retraction phase, dFR, along with standard deviations, for all tested materials in tests with the device in a mobile (**a**) and stationary (**b**) holder.

**Figure 8:**
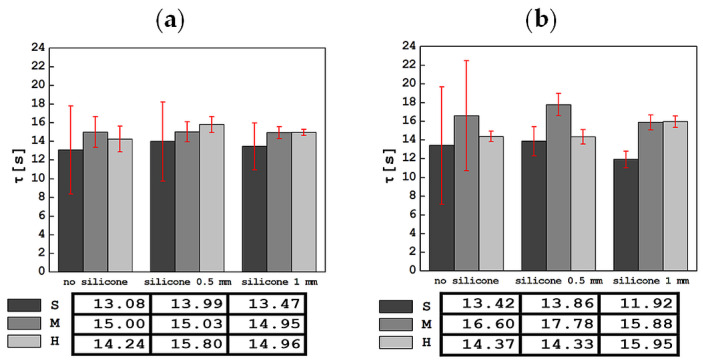
Comparison of averaged values of relaxation time τ, along with standard deviations for all tested materials in tests with the apparatus in a mobile (**a**) and stationary holder (**b**).

The results of the tests carried out using an indentation device in a mobile and stationary holder are shown in Figure 4, Figure 5, Figure 6, Figure 7 and Figure 8. The subjects of the comparison are the average values and standard deviations of the dFI, FImax, FRmax, dFR and τ descriptors, which were defined in Section 2 and found to be sensitive to changes in the properties of the tested materials. In each of the graphs, the results are presented in three groups: for S, M and H foam phantoms without an outer layer of silicone (marked “no silicone” in the graphs) and for the same foams with an added layer of silicone with a thickness of 0.5 mm (“silicone 0.5 mm” in the graphs) and 1 mm (“silicone 1 mm” in the graphs).

A comparison of the average values of the slope of the curve during indentation, dFI, along with the standard deviation, is shown in Figure 4. Adding a silicone layer or increasing its thickness maintains the increase in dFI in the groups, identically to the stiffness of the foams themselves (S, M, H). In most cases, higher dFI values were observed from tests with the device in a stationary holder than in a mobile one.

The lowest dFI values were measured for S foam alone and were 0.06 ± 0.01 N/s in stationary tests and 0.08 ± 0.01 N/s in mobile tests, respectively. Adding a layer of silicone with a thickness of 0.5 or 1 mm increased this value significantly. The highest values of the curve slope were recorded for the hardest foam (H) with a 1 mm layer of silicone and were 1.30 ± 0.05 N/s for the measurement in the mobile holder and 1.43 ± 0.02 N/s for the tests in the stationary holder.

The average values of the maximum force in the indentation phase, FImax, along with the standard deviations, are presented in Figure 5. Adding a silicone layer or increasing its thickness does not change the qualitative relationship resulting from the stiffness of the S, M and H foams. As expected, the lowest force values were obtained for the S foam without silicone layers. For tests in a stationary holder, FImax was 0.38 ± 0.02 N, and for tests in a mobile holder, 0.69 ± 0.02 N. The highest FImax values were recorded for the H foam with a 1 mm silicone layer, and in stationary tests, it was 14.61 ± 0.27 N and for tests in a mobile holder, 14.01 ± 0.53 N.

The average values of the maximum force in the retraction phase, FRmax, along with the standard deviations of this descriptor, are shown in Figure 6. It is worth noting that the values of this parameter are much lower than for FImax, regardless of whether the tested phantom had a silicone layer or not. However, the upward trend for this parameter was maintained. In this case, the lowest FRmax value of 0.25 ± 0.06 N was recorded for the S foam without a silicone layer, and the highest value of 13.62 ± 0.24 N for the H foam with 1 mm thick silicone, in tests with a stationary handle. For tests in the mobile holder, the lowest value was 0.48 ± 0.05 N for the S foam only, and the highest value was 12.97 ± 0.36 N for the H foam with the thickest silicone layer, similarly to the stationary holder.

The average values of the slope of the force versus time curve in the indenter retraction phase, dFR, are shown in Figure 7. Similarly to dFI, adding a silicone layer or increasing its thickness does not change the qualitative relationship resulting from the stiffness relationship of the S, M and H foams. For most tests, the dFR parameter determined based on the results obtained in the stationary holder had slightly higher values than those determined in the mobile holder. The lowest absolute slope of the curve during indenter retraction was 0.07 ± 0.01 N/s for the S foam without silicone, and the highest was 1.45 ± 0.01 N/s for the H foam with 1 mm silicone in the test with a stationary handle. The situation is similar for the case of tests in the mobile holder: 0.08 ± 0.01 N/s for S foam without silicone, and 1.32 ± 0.08 N/s for H foam with 1 mm of silicone.

As a result of approximating the dependence of force on time F(t) in the range from 10 to 60 s with an exponential curve, the relaxation time τ was determined and the average values of this parameter along with their standard deviations are presented in Figure 8. The value of the relaxation time for phantoms S (including with silicone) is systematically lower than for both other phantoms, both in tests with a mobile and stationary holder.

The results of testing phantoms with thinner and thicker silicone layers do not show a systematic effect of the silicone thickness or foam stiffness on the τ parameter. The results of the relaxation time obtained in tests with a stationary handle show a greater dispersion of values than in tests with a mobile handle. The lowest value of τ was 11.92 ± 0.88 s for S foam with a 1 mm thick silicone layer, and the highest was 17.78 ± 1.20 s for M foam with a 0.5 mm thick silicone layer. These values were slightly different in the mobile handle tests; here, the lowest value of 13.08 ± 4.71 s was recorded for S foam only, while the highest, for H foam with a 0.5 mm thick silicone layer, was 15.80 ± 0.86 s. It is worth noting that, for the mobile holder, adding silicone to each foam gradually reduced the size of the standard deviation.

## 4. Discussion

In this study, three cylindrical foam phantoms and the same phantoms with a 0.5 or 1 mm silicone layer were tested. Knowledge from testing such materials can be valuable in interpreting patient test results, especially in the case of tissue with lymphedema. Foam S was to imitate tissue with edema, M—healthy tissue and H—tissue in the phase in which fibrosis appears. Silicone layers were to imitate skin, and the criteria for selecting this material were thickness and stiffness, similar to the parameters of human skin.

**Figure 9:**
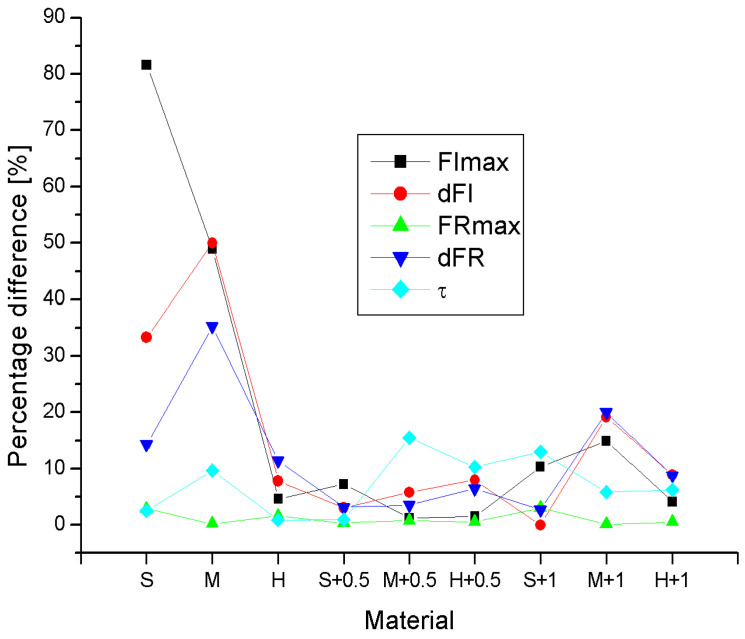
Comparison of percentage differences in descriptors for all studied phantoms determined using the indentation method with a mobile and stationary handle. Foam materials are marked S, M, H, materials with a 0.5 mm silicone layer are marked S + 0.5, M + 0.5 and H + 0.5 and materials with a 1 mm layer are marked S + 1, M + 1, H + 1.

Taking the results of the indentation tests carried out with a stationary set as reference results, the values of relative percentage differences in descriptors determined with the mobile set were determined according to the formula:

(1) $$RD=\left|\frac{Vm-Vs}{Vs}\right|\cdot 100\%$$

where *Vm* and *Vs* are the average values of the descriptors determined by the mobile and stationary sets, respectively, which are given in the tables in Figure 4, Figure 5, Figure 6, Figure 7 and Figure 8. The *RD* values for all nine tested materials are summarized in Figure 9. It can be noticed that the greatest discrepancies in the results obtained using the mobile and stationary handle (RD at the level of several dozen percent) concerned the FIMax, dFI and dFR parameters for the S and M foam samples. This is probably related to the fact that the foams are heterogeneous (noticed in compression measurements, see Table 1) and the lack of a silicone layer tones down this heterogeneity. Also contributing to the discrepancy may be the greater difficulty in correctly positioning the downhole apparatus in relation to the sample in the absence of silicone due to the softness of the material.

Comparing the descriptor values for all materials, it was found that the smallest differences in measurement results were visible for FRmax. This is not surprising considering the fact that this is the parameter least influenced by the uncertainty of measurement of values changing over time, because FRmax is measured after 60 s of load (beginning of retraction, approx. end of relaxation phase). When comparing the results in groups, i.e., for foams without silicone and with different silicone layers, the best agreement of the descriptors was obtained for foams with a 0.5 mm silicone layer. This is probably the result of the stabilizing effect of the silicone layer on stiffness, in particular by limiting the role of local inhomogeneities on the stiffness of the foams, which is significant even on the scale of samples tested in compression tests on a testing machine (see Table 1). However, increasing the thickness of the silicone layer does not enhance this effect.

To assess the usefulness of the results of measuring the maximum force value FImax of foams tested using the indentation method (tables in Figure 5), the Pearson correlation coefficient FImax with the Young’s modulus determined from the uniaxial compression test (see Table 1) was determined. The correlation coefficient is 0.965 and 0.999 for the test results with the mobile and stationary handle, respectively. The same analysis could be used for FRmax forces; however, due to the fact that the maximum values of the force of a one-minute compression test were measured (times similar to those in indentation tests) according to the ISO 844 standard [44], there is greater justification for comparing the stiffness results with FImax.

**Figure 10:**
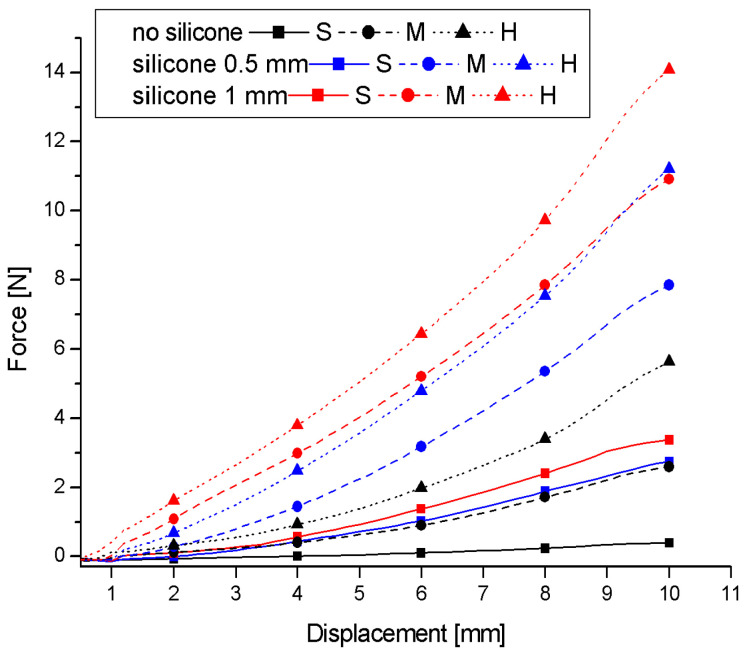
Representative force–displacement curves from the first period of the indentation test for all tested soft-material phantoms.

Very important information about the stiffness of materials tested by the indentation method is contained in the force–displacement relationship during the indentation phase. Figure 10 shows representative curves, selected from six tests on each phantom, of the dependence of the force on the displacement of the indenter in the indentation phase (first 10 s of the test). The results are from measurements with the indenter mounted on a stationary handle. In each case, a deviation of the dependence from a straight line is observed in most of the indentation ranges, characteristic of the elastic stiffening of the material. Only in the final phase do the trends change. Adding a layer of silicone to each of the foams increases the stiffness of the system but does not significantly change the nature of the force–displacement relationship. The increase in the stiffness of the system with a layer of silicone results from the fact that the silicone exerts additional resistance to the indenter.

Numerous publications devoted to indentation research (see e.g., [45,46]) attempt to use the results of the force response to an indentation of a material sample in the form of a homogeneous layer to estimate the uniaxial stiffness based on the Hayes equation:

(2) $$E=\frac{F\cdot \left(1-\nu^{2}\right)}{2a\cdot u_{z}\kappa }$$

where *F* is the force acting on the indenter of radius *a* when embedded in the material *u_z_*; *ν* is the Poisson’s ratio; and *κ* is a scaling factor depending on the relationship between the radius of the indenter and the thickness of the tested sample and takes values not less than one. If we adopt Formula (2) to calculate the Young’s modulus of S, M, H foams for which FImax forces were determined in a stationary holder, with a depth of 1 cm, assuming the Poisson number equal to 0 and a scaling factor equal to 1, we will obtain the following values of the E modulus: 3.4, 23.8 and 53.6 kPa. Comparing these values with the average results of uniaxial compression, 18.32, 46.44 and 85.72 kPa (Table 1), we see that except for the most flexible foam, the results are of the same order but differ significantly. Increasing the value of the Poisson’s ratio and the scaling factor will reduce the value of the Young’s modulus estimated based on the Hayes formula, and thus increase the discrepancy with the values determined in the uniaxial compression test. The above results confirm the existence of discrepancies between the stiffness moduli of soft materials determined using different measurement techniques, which were also noticed in other works, e.g., [46,47,48]. Looking for the reasons for this state of affairs, it seems that one of the reasons may be the cylindrical shape of the samples tested using the indentation method and the associated different distribution of stresses and strains than in the case of half-spaces or layers for which the Hayes formula is used. The cylindrical geometry causes the force response for a given displacement of the indenter to be correspondingly smaller, and therefore, compared to a flat material, the response is as if the material were more elastic. However, separate experimental or theoretical studies based on simulations would be necessary to establish the relationship corresponding to the cylindrical geometry of samples in indentation tests.

A more advanced problem is the mathematical description for a non-homogeneous material, explaining the influence of foam type and silicone thickness on the force–displacement relationship. In addition to many numerical models using the finite element method, analytical relationships are particularly interesting from a practical point of view. A group of 10 easily adaptable analytical models relating the force F to the indenter displacements u_z_ or their appropriate increments (*dF*, *du_z_*) can be found in the works [49,50], in which the moduli-perturbation method was used to obtain these models. A comparison of the prediction results of these models with the help of codes written in the Matlab environment allowed us to extract a model that relatively well approximates the results of our experimental phantom studies in the form:

(3) $$dF=4a{\left[\left[1-\nu_{s}+(\nu_{s}-\nu_{f})I_{1}\right]\left[\frac{1-I_{0}}{\mu_{s}}+\frac{I_{0}}{\mu_{f}}\right]\right]}^{-1}du_{z}$$

where *ν_s_*, *μ_s_* and *ν_f_*, *μ_f_* denote the Poisson’s ratio and the shear modulus of the foam (substrate) and the silicone layer (film), respectively, and *I*_0_ and *I*_1_ are functions of the parameter *ξ* = *h*/*a*, i.e., the quotient of the layer thickness h and the indenter radius a [50]:

(4) $$\begin{array}{l} I_{0}=\frac{2}{\pi }\arctan\xi +\frac{\xi }{\pi }\ln\frac{1+\xi^{2}}{\xi^{2}}\;;\;\\ I_{1}=\frac{2}{\pi }\arctan\xi +\frac{1}{2\pi (1-\nu )}\left[(1-2\nu )\xi \ln\frac{1+\xi^{2}}{\xi^{2}}-\frac{\xi }{1+\xi^{2}}\;\right] \end{array}$$

where *ν* is assumed to be the value of *ν_f_*.

**Figure 11:**
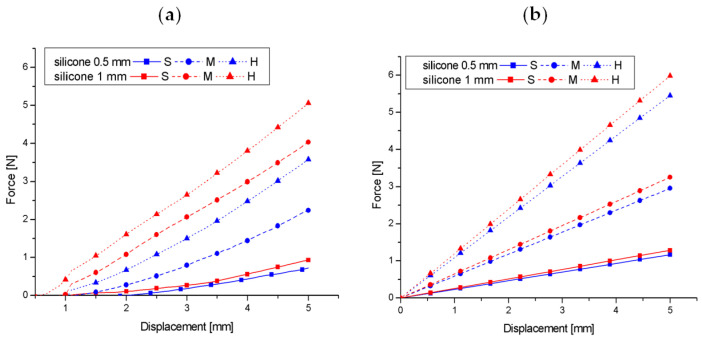
Comparison of the force–displacement relationship from the indentation test in the range of 0–5 mm based on measurements for foam phantoms with a silicone layer (**a**) and the analytical model described by Formula (3) (**b**).

Figure 11a,b compare the experimental results of foam phantoms with 0.5 and 1 mm silicone layers up to a 5 mm indentation (half of the range shown in Figure 10 because nonlinearities are clearly visible further on) with the predictions of model (3) in which the Young’s moduli for foams (S, M, H) were assumed according to Table 1, the Young’s modulus of silicone estimated from uniaxial tension was assumed to be 1 MPaand *ν_s_*= 0 and *ν_f_* = 0.5 were assumed, while the shear moduli μ_s_, μ_f_ were calculated from the formula for an isotropic material *μ* = *E*/2 (1 + *ν*). Taking into account the high heterogeneity of the tested foams and the fact that the tests were conducted on the curved side-surfaces of the phantoms, while the relationship (3) was derived for flat materials, one can notice a relatively good quantitative agreement of the model (3) with the experimental results. The greatest discrepancies occur for M foam with a 1 mm silicone layer and H foam with a 0.5 mm layer.

Taking into account the results of the above comparison, it is justified to use model (3) or other analytical models from the works [49] or [50] and the results of indentation tests in further work to develop methods for identifying the stiffness of the components of the soft substrate/layer composition.

**Figure 12:**
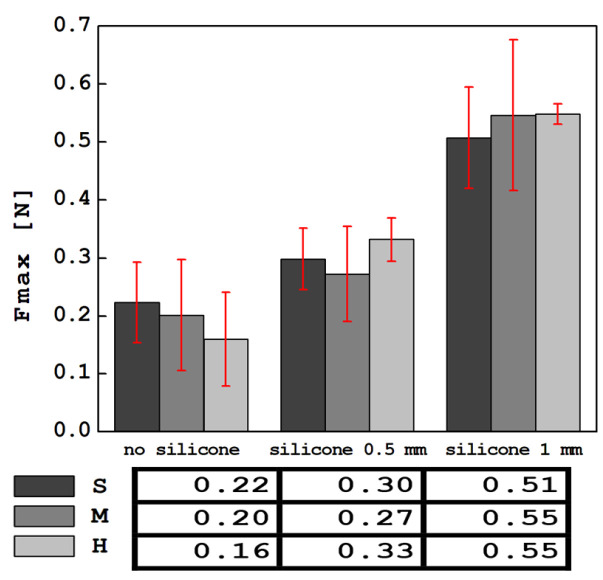
Summary of average force values from indentation measurements using the SkinFibroMeter device.

The collected results of the average values of the maximum force, Fmax, determined from indentation tests with an indentation apparatus held in the hands of the SkinFibroMeter operator—obtained for all tested samples—are shown in Figure 12. The lowest force values of Fmax were read for materials without a silicone layer, and the highest after adding a 1 mm thick silicone layer. The Fmax force values for S, M and H foams without silicone and with a 0.5 mm and 1 mm silicone layer do not correlate positively with the stiffnesses of the foams (Table 1) or the FImax values for the foams themselves. It is worth noting that the significant dispersion of Fmax results is represented by the standard deviation, which is comparable to the observed differences in Fmax in each group. In turn, the correlation of the Fmax and FImax values for a specific foam (without silicone, with 0.5 mm of silicone and 1 mm of silicone) is different. The Pearson coefficients for the S, M and H foams in these groups are 0.989, 0.975 and 0.99, respectively. Taking the above into account, it can be concluded that SkinFibroMeter measurements, which perform shallow penetration, are suitable for estimating the stiffness of materials in which it results from the presence of a surface layer, such as skin, in the system of soft tissue layers. A comparison and discussion of the results obtained using the SkinFibroMeter and several other techniques, as well as for other soft materials, confirming the above-noted discrepancy in the results obtained using different measurement methods, can be found in [51,52].

Finally, it is worth emphasizing that the deep indentation method used in the laboratory, compared to most traditional methods of testing the mechanical properties of materials (compression, elongation, bending), avoids the need to cut samples from soft materials (including two-phase materials), which often generates serious technical problems.

No information was found in the literature on the porosity and density of the tested materials and their relationship with stiffness. There are also no data on the observed spatial macro-heterogeneity of their stiffness. In future studies, it is worth devoting more space to these issues, in particular the relationship of the foam structure with elastic and viscous properties.

## 5. Conclusions

Indentation tests carried out using an indenter with an area of 1 cm^2^ inserted to a depth of 1 cm and withdrawn after approximately 60 s showed that foam materials alone and with a silicone layer can be effectively characterized by the descriptors determined from these tests. The values of the maximum forces during indentation, FImax, and retraction, FRmax, of the indenter, as well as the slope of the indentation curves, dFI, and retraction, dFR, are suitable for this purpose. There was no significant effect of the foam stiffness or additional silicone layer on the relaxation time values, τ. However, the influence of internal viscosity present in the case of S foam on the reduction in the relaxation time value is noticeable. The above features of the deep indentation method distinguish it from the shallow indentation method using the SkinFibroMeter (height and radius of the indenter = 1.25 mm), which mainly reflects the stiffness of the silicone layer and not the foam itself.

A comparison of the results (averages and spreads) obtained for the tests using different indentation apparatus holders shows that the repeatability of the tests performed in the mobile and stationary holders is comparable. The mobile handle is slightly less stable, but considering the heterogeneity of foam materials, this does not play an important role in testing such materials. It is also worth paying attention to the fact that an indentation apparatus mounted on a holder (mobile or stationary) limits the dispersion of the measurement results compared to a device held in the operator’s hand (results from the SkinFibroMeter device). Summarizing the results obtained from tests on foam phantoms, it can be stated that the use of the deep indentation method and the use of handles for mounting the indentation apparatus affect the objectivity of the indentation tests.

The results of the conducted experimental studies show that complementary theoretical studies describing or explaining the observed trends are necessary. Simple models that allow identifying the properties of homogeneous and non-homogeneous soft materials based on indentation test data would be particularly useful. The attempts to use the Hayes model or the layer-on-substrate model included in the work do not achieve this goal but may be useful in its implementation.

## Figures and Tables

**Table 1:** Young’s modulus for foams determined in compression tests from 5 samples.

	Young’s Modulus of the Samples E_d_ [kPa]	Mean
S foam	17.7/13.2/27.0/18.4/15.3	18.32
M foam	34.4/22.5/70.4/71.1/33.8	46.44
H foam	86.7/21.9/102/112/106	85.72

## Data Availability

The raw data supporting the conclusions of this article will be made available by the authors on request.
